# Supplementary material for: Non-Scanning Fiber-Optic Near-Infrared Beam Led to Two-Photon Optogenetic Stimulation In-Vivo
Source: PLoS One. 2014 Nov 10;9(11):e111488. doi: 10.1371/journal.pone.0111488 (PMC4226470; doi:10.1371/journal.pone.0111488)
Supplement: Figure S1 — Fiber-optic two-photon optogenetic stimulation set up and characterization. (a) Patch clamp set up for FO-TPOS. FSL: Tunable Ti: Sapphire Laser; BE: Beam Expander; S: Shutter; NDF: Neutral density filter; L: Lens for fiber coupling; FL: Fluorescence excitation source; Ex: Excitation Filter; Em: Emission Filter; MO: Microscope Objective; CL: Condenser lens; DM: Dichroic Mirror; M: Mirror; HL: Halogen Lamp. (b) Variation of the width of the ultrafast pulse from the multimode fiber as a function of laser power. (c) In-vivo electrophysiology set up for FO-TPOS. (d) Typical transverse beam profile emanating from the multimode fiber; (e) Time-lapse (1 sec) intensity profiles (in different colors) along a line drawn across the beam profile shown in d. (DOCX) [file pone.0111488.s001.docx]

**M**

**a**

**Ex**

**CL**

**NDF**

**Fiber**

**L**

**BE**

**Amplifier**

**DM**

**Em**

**PC**

**CCD**

**S**

**FL**

**HL**

**FSL**

**MO**

|   **d**  **e** | **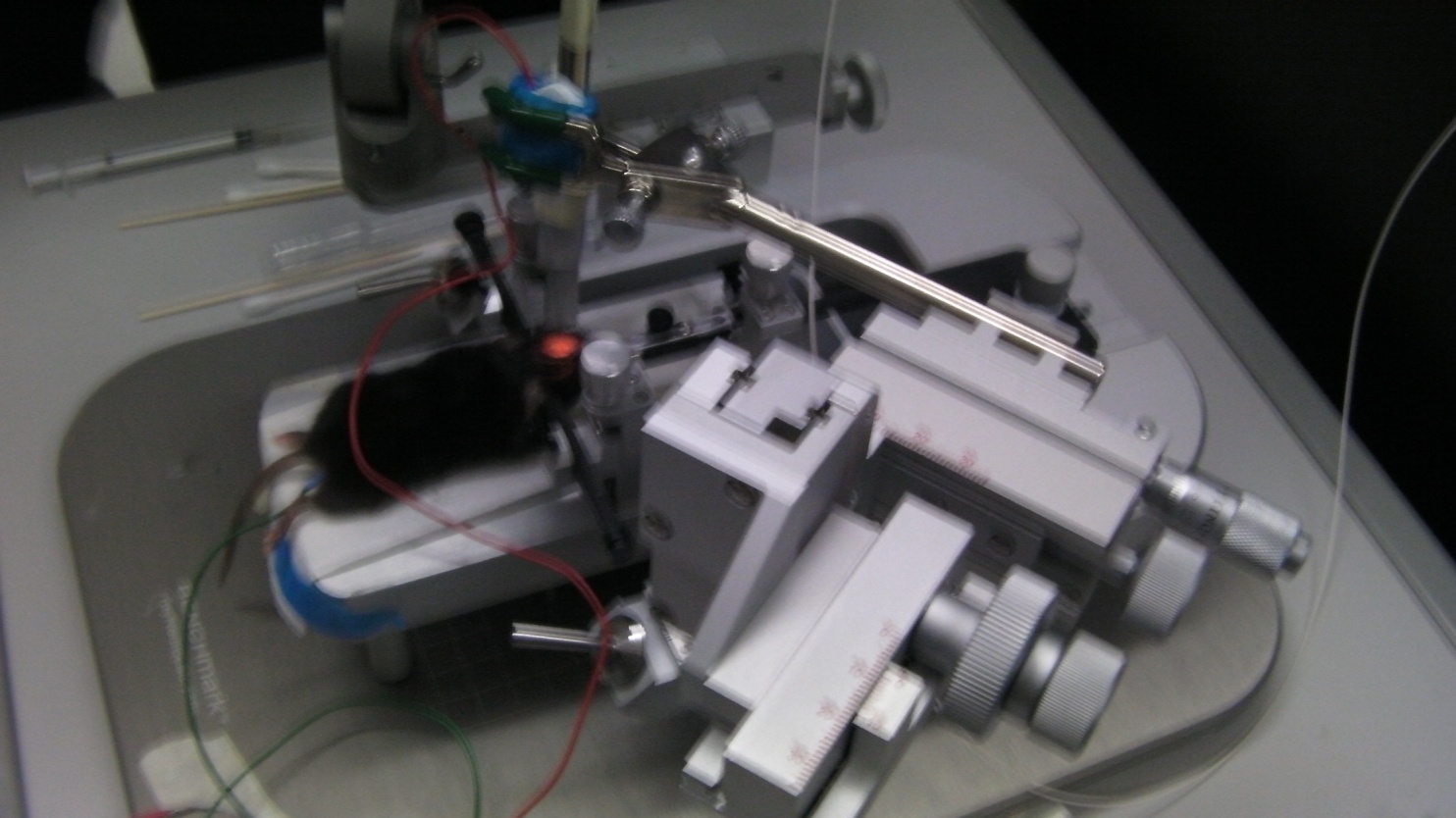**  **Optical fiber**  **Electrode** |
| --- | --- |
| 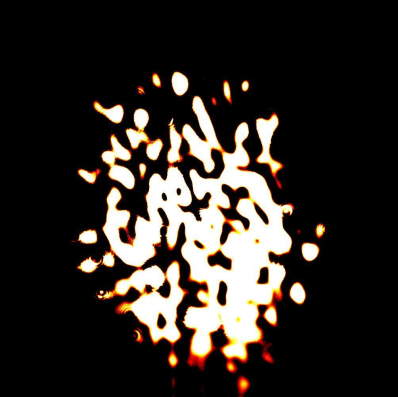 |  |

**c**

**b**

**Figure S1: Fiber-optic two-photon optogenetic stimulation set up and characterization.** (a) Patch clamp set up for FO-TPOS. FSL: Tunable Ti: Sapphire Laser; BE: Beam Expander; S: Shutter; NDF: Neutral density filter; L: Lens for fiber coupling; FL: Fluorescence excitation source; Ex: Excitation Filter; Em: Emission Filter; MO: Microscope Objective; CL: Condenser lens; DM: Dichroic Mirror; M: Mirror; HL: Halogen Lamp. (b) Variation of the width of the ultrafast pulse from the multimode fiber as a function of laser power. (c) *In-vivo* electrophysiology set up for FO-TPOS. (d) Typical transverse beam profile emanating from the multimode fiber; (e) Time-lapse (1sec) intensity profiles (in different colors) along a line drawn across the beam profile shown in d.
