# Supplementary material for: Non-Scanning Fiber-Optic Near-Infrared Beam Led to Two-Photon Optogenetic Stimulation In-Vivo
Source: PLoS One. 2014 Nov 10;9(11):e111488. doi: 10.1371/journal.pone.0111488 (PMC4226470; doi:10.1371/journal.pone.0111488)
Supplement: Figure S2 — Estimation of two photon absorption cross section for ChR2. (a–b) Different traces of photocurrent in ChR2-sensitized HEK cells induced by fiber-optic near-infrared stimulation using 250 fs pulsed laser beam (870 nm, 80 MHz) at average intensity of 0.02 mW/µm2. The fitted data (using Eq. 1) is overlaid (red traces) over the measured photocurrent. The fitted parameters τ1 and τ2 are used to calculate the two-photon absorption cross section. (DOCX) [file pone.0111488.s002.docx]

|   **b**  **a** |
| --- |
| **Normalized current** |

**Figure S2.** **Estimation of two photon absorption cross section for ChR2**. (a, b) Different traces of photocurrent in ChR2-sensitized HEK cells induced by fiber-optic near-infrared stimulation using 250 fs pulsed laser beam (870 nm, 80 MHz) at average intensity of 0.02 mW/µm^2^. The fitted data (using Eq. 1) is overlaid (red traces) over the measured photocurrent. The fitted parameters *τ_1_* and *τ_2_* are used to calculate the two-photon absorption cross section.
