# Supplementary material for: Non-Scanning Fiber-Optic Near-Infrared Beam Led to Two-Photon Optogenetic Stimulation In-Vivo
Source: PLoS One. 2014 Nov 10;9(11):e111488. doi: 10.1371/journal.pone.0111488 (PMC4226470; doi:10.1371/journal.pone.0111488)
Supplement: Figure S3 — Wavelength-dependent fiber-optic two-photon optogenetic stimulation in-vivo . (a) Raw spiking activity during in-vivo electrophysiology subsequent to fiber-optic two-photon optogenetic stimulation (FO-TPOS) at different wavelengths (average incident laser power: 80 mW, 5 Hz), (b) Peak voltage vs. wavelength of in-vivo FO-TPOS at two different laser power densities, (c) Firing rate (spikes per second) vs. wavelength of in-vivo FO-TPOS. (DOCX) [file pone.0111488.s003.docx]

| **800 nm**  **a**  **875 nm**  **0.05 mV**  **150 ms**    **900 nm**  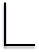  **c** | |
| --- | --- |
|   **b**  **c**  **e** |  |

**Figure S3. Wavelength-dependent fiber-optic two-photon optogenetic stimulation *in-vivo*.** (a) Raw spiking activity during in-vivo electrophysiology subsequent to fiber-optic two-photon optogenetic stimulation (FO-TPOS) at different wavelengths (average incident laser power: 80 mW, 5Hz), (b) Peak voltage vs. wavelength of in-vivo FO-TPOS at two different laser power densities, (c) Firing rate (spikes per second) vs. wavelength of in-vivo FO-TPOS.
