# Supplementary material for: Non-Scanning Fiber-Optic Near-Infrared Beam Led to Two-Photon Optogenetic Stimulation In-Vivo
Source: PLoS One. 2014 Nov 10;9(11):e111488. doi: 10.1371/journal.pone.0111488 (PMC4226470; doi:10.1371/journal.pone.0111488)
Supplement: Figure S4 — Fiber-optic two-photon optogenetic stimulation of negative control. Direct two-photon illumination of micropipette-electrode separated by 1 mm (a) no media and (b) in phosphate buffer saline. (c) Recording from micropipette-electrode during two-photon illumination in dead brain. (DOCX) [file pone.0111488.s004.docx]

| ****  **a**  **b**  **c** |
| --- |
| **** |
|  |

**Figure S4. Fiber-optic two-photon optogenetic stimulation of negative control.** Direct two-photon illumination of micropipette-electrode separated by 1mm (a) no media and (b) in phosphate buffer saline. (c) Recording from micropipette-electrode during two-photon illumination in dead brain.
