# Supplementary material for: Non-Scanning Fiber-Optic Near-Infrared Beam Led to Two-Photon Optogenetic Stimulation In-Vivo
Source: PLoS One. 2014 Nov 10;9(11):e111488. doi: 10.1371/journal.pone.0111488 (PMC4226470; doi:10.1371/journal.pone.0111488)
Supplement: Figure S5 — Monte Carlo simulation of light propagation in two-layered cortex for comparison of fiber-optic two-photon optogenetic stimulation vs fiber-optic single-photon optogenetic stimulation. Effects of numerical aperture (NA) of optical fiber (0.15, 0.22, and 0.3) and laser beam power (1, 5, and 50 mW) on XZ-distribution of power density (W/cm2) shown for near-infrared (870 nm) as well as blue (470 nm). Parameters for the MC simulation are listed in Table S1. (DOCX) [file pone.0111488.s005.docx]

**Figure S5.** **Monte Carlo simulation of light propagation in two-layered cortex for comparison of fiber-optic two-photon optogenetic stimulation vs fiber-optic single-photon optogenetic stimulation.** Effects of numerical aperture (NA) of optical fiber (0.15, 0.22, and 0.3) and laser beam power (1, 5, and 50 mW) on XZ-distribution of power density (W/cm^2^) shown for near-infrared (870 nm) as well as blue (470 nm). Parameters for the MC simulation are listed in Supplementary Table 1.
