# Supplementary material for: Non-Scanning Fiber-Optic Near-Infrared Beam Led to Two-Photon Optogenetic Stimulation In-Vivo
Source: PLoS One. 2014 Nov 10;9(11):e111488. doi: 10.1371/journal.pone.0111488 (PMC4226470; doi:10.1371/journal.pone.0111488)
Supplement: Figure S6 — Intensity and depth-dependent fiber-optic two-photon optogenetic stimulation. Variation of peak amplitude of two-photon activated electrical recording as a function of depth at different power densities (a) 870 nm, (b) 900 nm. Histogram of peak-amplitude as a function of power densities of in-vivo fiber-optic TPOS at different depths. (c) 850 nm, (d) 870 nm. (DOCX) [file pone.0111488.s006.docx]

|   **a** |   **b** |
| --- | --- |
|   **850 nm**  **d**  **c** |   **870 nm** |

**Figure S6. Intensity and depth-dependent** **fiber-optic two-photon optogenetic stimulation.** Variation of peak amplitude of two-photon activated electrical recording as a function of depth at different power densities (a) 870 nm, (b) 900 nm. Histogram of peak-amplitude as a function of power densities of *in-vivo* fiber-optic TPOS at different depths*.* (c) 850 nm, (d) 870 nm*.*
