# Supplementary material for: Non-Scanning Fiber-Optic Near-Infrared Beam Led to Two-Photon Optogenetic Stimulation In-Vivo
Source: PLoS One. 2014 Nov 10;9(11):e111488. doi: 10.1371/journal.pone.0111488 (PMC4226470; doi:10.1371/journal.pone.0111488)
Supplement: Figure S7 — Comparison of microscopic two-photon setup with defocused and focused fiber-optic two-photon optogenetic stimulation. (DOCX) [file pone.0111488.s007.docx]

**Microscopic two-photon**

**Fiber-optic focused two-photon**

**Fiber-optic defocused two-photon**

**Figure S7.** **Comparison of microscopic two-photon setup with defocused and focused fiber-optic two-photon optogenetic stimulation.**
