# Supplementary material for: Non-Scanning Fiber-Optic Near-Infrared Beam Led to Two-Photon Optogenetic Stimulation In-Vivo
Source: PLoS One. 2014 Nov 10;9(11):e111488. doi: 10.1371/journal.pone.0111488 (PMC4226470; doi:10.1371/journal.pone.0111488)
Supplement: Table S1 — Parameters for Monte Carlo simulation. Beam power is 5 mW, diameter is 60 µm and NA is 0.15. Where, µa = Absorption coefficient; µs = Scattering coefficient; g = anisotropy factor; n = refractive index, and d = thickness of cortical layers. (DOCX) [file pone.0111488.s008.docx]

**Supporting Information**

| Wavelength | Layer | µ_a_ (mm^-1^) | µ_s_(mm^-1^) | g | n | d (mm) |
| --- | --- | --- | --- | --- | --- | --- |
| 470 nm | Grey matter | 0.4 | 10.3 | 0.87 | 1.36 | 0.5 |
|  | White matter | 0.36 | 43 | 0.78 | 1.38 | 2.5 |
| 870 nm | Grey matter | 0.02 | 7.5 | 0.90 | 1.36 | 0.5 |
|  | White matter | 0.06 | 31 | 0.88 | 1.38 | 2.5 |

**Table S1.** **Parameters for Monte Carlo simulation.** Beam power is 5 mW, diameter is 60 µm and NA is 0.15. Where, μ_a_ = Absorption coefficient; μ_s_ = Scattering coefficient; g = anisotropy factor; n = refractive index, and d= thickness of cortical layers.
